# Distributed practice. The more the merrier? A randomised bronchoscopy simulation study

**DOI:** 10.3402/meo.v21.30517

**Published:** 2016-05-10

**Authors:** Anne Sofie Bjerrum, Berit Eika, Peder Charles, Ole Hilberg

**Affiliations:** 1 Department of Respiratory Diseases, Aarhus University Hospital, Aarhus, Denmark; 2 Rector's Office, Aarhus University, Aarhus, Denmark; 3 Centre for Health Sciences Education, Aarhus University, Aarhus, Denmark

**Keywords:** medical education, simulation, training methods, curriculum design, bronchoscopy

## Abstract

**Introduction:**

The distribution of practice affects the acquisition of skills. Distributed practice has shown to be more effective for skills acquisition than massed training. However, it remains unknown as to which is the most effective distributed practice schedule for learning bronchoscopy skills through simulation training. This study compares two distributed practice schedules: One-day distributed practice and weekly distributed practice.

**Method:**

Twenty physicians in training were randomly assigned to one-day distributed or weekly distributed bronchoscopy simulation practice. Performance was assessed with a pre-test, a post-test after each practice session, and a 4-week retention test using previously validated simulator measures. Data were analysed with repeated measures ANOVA.

**Results:**

No interaction was found between group and test (*F*(4,72) <1.68, *p*>0.16), except for the measure ‘percent-segments-entered’, and no main effect of group was found for any of the measures (*F*(1,72)< 0.87, *p*>0.36), which indicates that there was no difference between the learning curves of the one-day distributed practice schedule and the weekly distributed practice schedule.

**Discussion:**

We found no difference in effectiveness of bronchoscopy skills acquisition between the one-day distributed practice and the weekly distributed practice. This finding suggests that the choice of bronchoscopy training practice may be guided by what best suits the clinical practice.

Simulation-based training has become an integrated part of the training of many procedural and surgical skills because it offers students and physicians in training an opportunity to practise their skills in a safe and stress-reduced environment. To optimise the skills training laboratory experience, flexible and feasible training methods are needed. The scheduling of such practice is shown to influence skills acquisition in psychomotor as well as in cognitive domains; studies within these domains have shown that distributed practice is superior to massed practice for tasks like typing, ball toss, and second-language learning (1, 2). Massed practice refers to a practice schedule that allows no periods of rest in between practice, whereas distributed practice refers to a schedule where periods of practice are interspersed with periods of rest (3). The underlying mechanisms driving the beneficial effects of distributed practice are not well understood. Theoretical explanations for the effect of distributed practice relate these effects to the larger number of retrieval cues in distributed practice than in massed practice, which may facilitate recollection (4).

Within the context of medical clinical skills courses, only distributed practice schedules are relevant to examine as even short courses are variations of distributed practice. Several distributed practice schedules have been examined within medical procedural skills learning studies with diverse results. Verdaasdonk et al. (3) compared a one-day distributed practice schedule with a practice schedule distributed within three consecutive days for learning basic laparoscopic skills on a virtual reality simulator (3). They found that the latter practice was more effective than the former, but this superiority was noted only for the ‘time score’. Weekly and monthly practice schedules have also been studied. Moulton et al. (5) found a strong distributed practice effect of weekly distributed practice compared with one-day distributed practice (practice distributed within the same day) for learning microvascular anastomosis. Mitchell et al. (6) found a weekly distributed practice schedule and a monthly distributed practice schedule to be equally effective for learning microvascular anastomosis. Initial work on distributed practice schedules within medical simulation training has shown various beneficial effects of several distributed practice schedules ranging from one-day distributed practice to monthly distributed practice. Two reviews on the distributed practice effect within the psychomotor domain and the cognitive domain showed that the magnitude of the distributed practice effect found in previous studies was influenced by task type, task complexity, and the interval between practice sessions (1, 2). Previous medical procedural skills learning studies have focused on suturing tasks and basic hand–eye coordination tasks which differ much in terms of tasks and their complexity from those involved in bronchoscopy skills acquisition wherefore the optimal practice schedule for learning bronchoscopy remains unknown.

In the present study, we therefore compare the effectiveness of a weekly distributed practice schedule (a practice schedule with promising effects on skills acquisition within medical simulation training) with that of a one-day distributed practice schedule (the practice schedule of most clinical skills training courses) for learning bronchoscopy skills on a high-fidelity simulator.

## Method

### Study design

We conducted a randomised bronchoscopy simulation study on the effects of one-day distributed practice compared with weekly distributed practice.

### Participants

Participants included 20 PGY 1–PGY 3 residents from the Department of Respiratory Diseases at Aarhus University Hospital in Denmark. They were enrolled in the study from 2010 to 2012. Approximately 25 PGY 1–PGY 3 residents were employed at the department at that period. Exclusion criteria were previous experience with bronchoscopy simulators and experience with performing real-life bronchoscopy. Two residents declined to participate in the study. Each participant provided informed consent and was randomly assigned to one-day distributed practice or weekly distributed practice using a randomisation procedure with closed envelopes. The number of participants in each group was determined with a power calculation. Ten participants were enrolled in each group.

### The procedure

When performing a bronchoscopy, the airways are inspected with a flexible bronchoscope to the sub-segmental bronchus level. The scope is to be centred in the lumen to avoid wall collision and to avoid red-out (which occurs when the tip of the scope is pressed against the mucosa). Orientation is difficult and systematic inspection of the bronchial system is essential to secure a complete inspection and to reduce repeated sub-segmental bronchi inspection, thereby reducing procedure time and the risk of wall collisions and red-out.

### The simulator

The simulator is an Accutouch high-fidelity virtual reality simulator from CAE Healthcare^®^, Quebec, Canada. It consists of a proxy flexible bronchoscope, a robotic interface device, a computer with simulation software, and a monitor. The virtual patient responds to the trainee's instrumentation of the equipment with coughing, and the simulator provides force-feedback which further enhances the realism.

### Training

Before training, participants were introduced to the simulator equipment by an instructor. An atlas of the airways was handed out and studied by the participants, and they watched a 15-min instruction video available on the simulator.

**Fig. 1:**
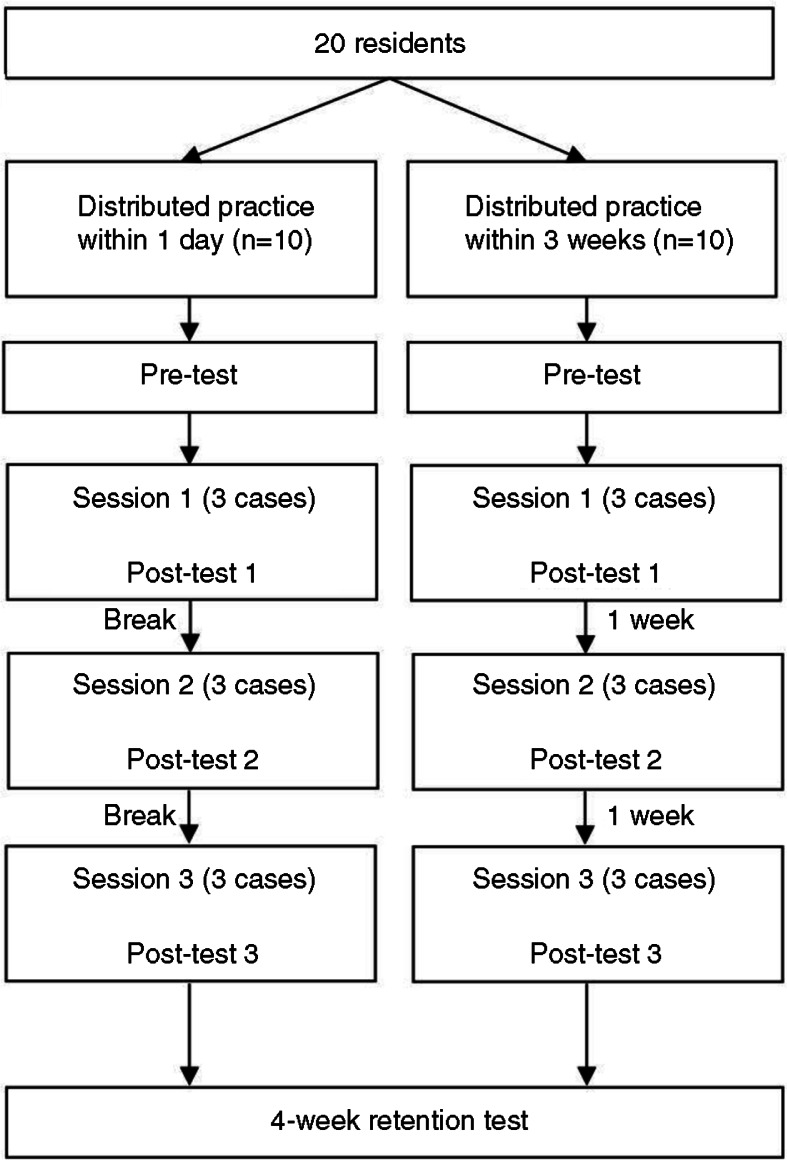
Study flow chart.

The training was divided into three sessions. Participants in the one-day distributed practice group received the three sessions within one day, and participants in the weekly distributed practice group attended the same three sessions within a period of 3 weeks, one session per week. Two major breaks (a coffee break and a lunch break) were scheduled within the programme for the one-day distributed practice group (see Fig. 1).

During each practice session, participants first watched the 15-min video available on the simulator; the video showed a consultant explaining and demonstrating how to navigate the bronchoscope. Thereafter, participants practised unsupervised on the same bronchoscopy simulator case three times. An instructor was present in the room, but no performance feedback was given to participants to avoid potential bias from an instructor. An atlas of the airways and a utility in the simulator software (road signs) naming the different segmental bronchi on the monitor were available for all participants throughout the training. A time limit of 20 min was allowed for each case. After completing each case, a case summary appeared on the monitor, presenting procedure time, wall collisions, time in red-out, and percent-segments-entered for the particular case performed.

### Assessment

To establish the initial skill level, participants completed a pre-test consisting of a simulator case that was not part of the training. Learning outcomes were assessed after each of the three training sessions with post-test 1, post-test 2, and post-test 3 using the same test-case as the pre-test. To assess how well skills were retained, participants were assessed 4 weeks after the last post-test with a retention test using the same test-case used for pre- and post-tests.

During testing, participants were instructed to perform the simulated bronchoscopy as quickly and thoroughly as possible, and no simulator utility and no atlas of the airways were available during testing.

Participants were tested with bronchoscopy simulator metrics. Validity evidence has been accumulated previously for these simulator measures in the form of construct validity evidence (7–9). These included ‘procedure time’, ‘percent-segments-entered’, ‘wall collisions’, and ‘red-out’. The combined measure ‘percent-segments-entered-per-minute’ was chosen as the primary outcome measure as we believe that it reflects bronchoscopy skills learning to a higher extent than ‘percent-segments-entered’ and ‘procedure time’ in isolation.

### Ethics

Participants provided informed consent and anonymity was guaranteed. As this study was conducted in a non-clinical setting, an exemption letter was issued from the Danish local ethical committee.

### Statistics

Data were analysed using STATA version 11.0 (Stata-Corp LP, College Station, TX, USA).

Data were log transformed in order to fulfil the assumptions necessary for using univariate repeated measures analyses of variance (ANOVA). Separate univariate repeated measures ANOVAs were applied on each variable with test (pre-test, post-test 1, post-test 2, post-test 3, and retention test) as within-subjects factor and group as between-subjects factor. Post-hoc analyses of group differences were Bonferroni corrected. A significance level of 0.01 was used as five pairwise comparisons were made on each variable. For all other analyses, a significance level of 0.05 was used.

## Results

Twenty physicians in training participated in the study. Pre-test, post-tests (1, 2, and 3), and 4-week retention tests were collected for all physicians in training.

**Fig. 2:**
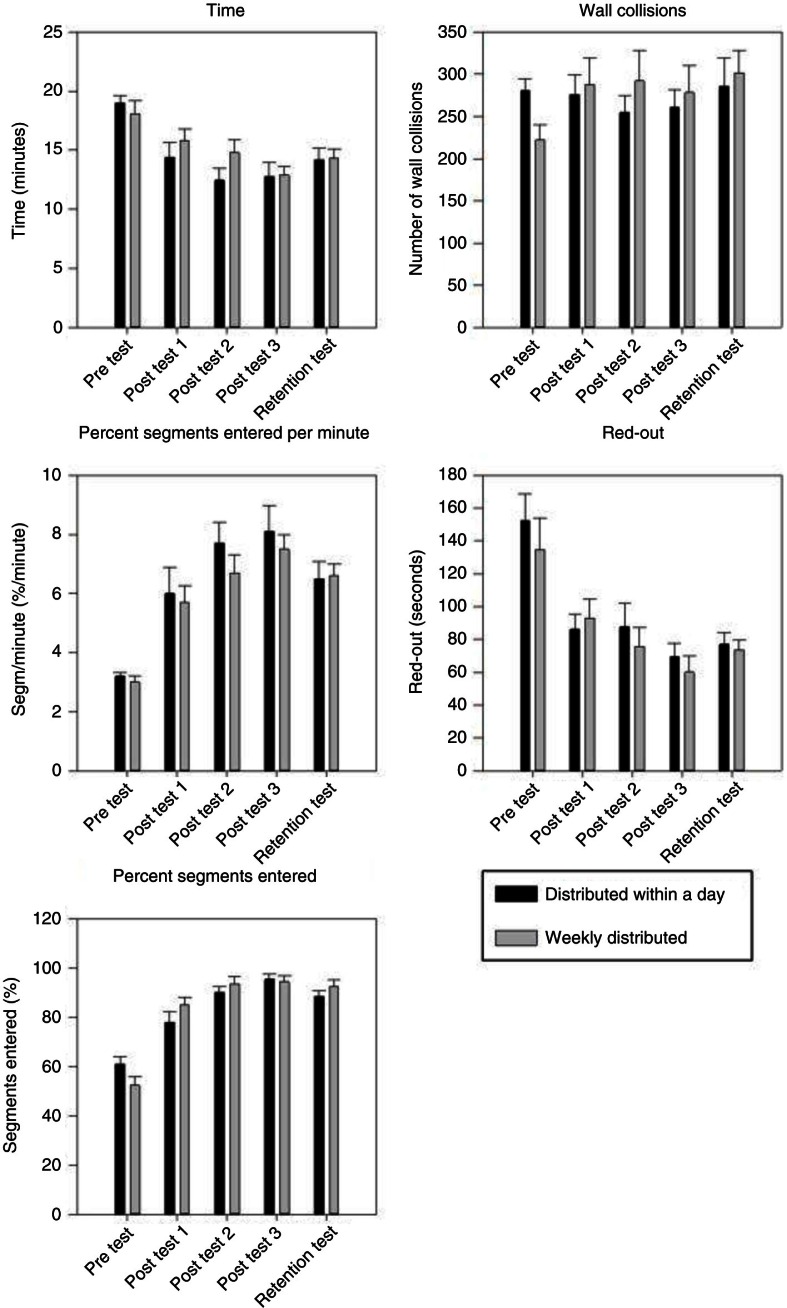
Simulator metrics test scores (mean+standard error) as a function of pre-test, post-test 1, post-test 2, post-test 3, and retention test for the daily distributed practice group and the weekly distributed practice group.

Test data for each variable (mean±standard error) are depicted in Fig. 2. The results of the repeated measures ANOVAs are outlined in Table 1.

**Table 1:** Statistical analyses from the repeated measures analyses of variance (ANOVA) on log-transformed measures from the pre-test, post-tests, and retention tests

	Test×group interaction	Group main effect	Test main effect
Segments/minute	*F*(4,72)=0.43, *p*=0.79	*F*(1,18)=0.03, *p*=0.87	*F*(4,72)=52.89, *p*<0.0001
Collisions	*F*(4,72)=1.68, *p*=0.16	*F*(1,18)=0.07, *p*=0.80	*F*(4,72)=0.76, *p*=0.55
Red-out	*F*(4,72)=0.37, *p*=0.83	*F*(1,18)=0.87, *p*=0.36	*F*(4,72)=13.95, *p*<0.0001
Time	*F*(4,72)=0.99, *p*=0.42	*F*(1,18)=0.00, *p*=0.95	*F*(4,72)=110.11, *p*<0.0001
Segments	*F*(4,72)=2.89, *p*=0.03	*F*(1,18)=0.11, *p*=0.75	*F*(4,72)=58.23, *p*<0.0001

A significant main effect of the test was found for all measures (*F*(2,72) > 11.11, *p*<0.0001) except for the variable ‘wall collisions’, which indicates a significant performance improvement from pre-test to post-test and retention test. No main effect of group was found for any of the variables (*F*(1,72)< 0.87, *p*>0.36), which indicates no difference in test scores between the two distributed practice conditions. Except for the variable ‘percent-segments-entered’, no interaction between group and test was found for any of the variables, that is, the learning curves were parallel.

Post-hoc analyses of the significant interaction between test and group for the measure ‘percent-segments-entered’ showed no significant differences between the two practice groups at any time point.

Post-hoc analyses of the significant main effect of test on the variables ‘percent-segments-entered-per-minute’, ‘percent-segments-entered’, ‘procedure time’, and ‘red-out’ showed a significant increase in test score from pre-test to the final post-test (post-test 3) (*p* < 0.001 for all variables) and retention test (*p* < 0.001 for all variables). Deterioration in performance from the final post-test (post-test 3) to retention test was seen for all variables, as shown in Fig. 2; however, the deterioration was not statistically significant for any of the variables.

## Discussion

In this study, we compared the effect of two different distributed practice schedules (one-day distributed practice and weekly distributed practice) on the acquisition of bronchoscopy skills. We found no difference in effectiveness of skills acquisition between one-day distributed practice and weekly distributed practice, that is, we found no additional effect by distributing the practice beyond a single day.

This result differs from the results reported in most previous studies on distributed practice within medical procedural and surgical skills training (3, 5, 10), which show that daily and weekly distributed practices are more effective for skills acquisition than one-day distributed practice. However, important differences exist between our study and previous studies, and these differences may have influenced the distributed practice effect. Most importantly, there are differences between the task types and the complexity of the tasks examined in previous studies and our study. The skills examined in previous surgical skills training studies were basic hand–eye coordination tasks in laparoscopy skills learning (3, 10) and suturing tasks in microvascular anastomosis training (5, 6). The tasks involved in basic laparoscopy skills learning and suturing contain a higher degree of psychomotor skills components than the tasks involved in performing and learning bronchoscopy skills. Furthermore, the orientation tasks and pattern recognition tasks involved in bronchoscopy skills acquisition are cognitively more complex than the basic hand–eye coordination tasks and suturing tasks. The influence of task type and task complexity on the distributed practice effect is discussed in a review by Donovan et al. (2) They argue that the strong distributed practice effect is limited to tasks that are low in overall complexity and mental requirements but high in physical requirements, such as ball tossing and typing (2). Thus, it is likely that the training of a task like microvascular suturing, which involves a high degree of psychomotor involvement but possibly a lower degree of overall complexity, benefits more from a weekly distributed practice schedule than does the training of bronchoscopy. Consequently, the results of our study support a previously proposed association: that the size of the distributed practice effect appears to depend on task type and task complexity as well as the length of time in between practice sessions (2, 11).

Our results were obtained in an unsupervised practice setting. Participants from both practice groups (both the one-day distributed practice group and the weekly distributed practice group) showed a relatively slow performance improvement from pre-test to post-test compared with other bronchoscopy simulation studies that included feedback (12, 13). The most important difference in the training set-up between these studies is the inclusion/exclusion of feedback and supervision. Thus, our results reveal the effects of training in an unsupervised training setting. Supervision and feedback is important in complex skills learning, and studies have consistently shown larger learning gains for practice curricula that include feedback than for those that do not (14–16). However, in our study the unsupervised practice setting was chosen to enhance the feasibility of a weekly distributed practice schedule. Furthermore, the unsupervised practice setting secured an experimental study set-up without the potential bias that may emanate from an instructor providing feedback.

Feasibility is an important feature to account for in the practice curriculum. In an unsupervised practice setting, the scheduling of practice involves only the physician in training. In such a setting, a weekly distributed practice schedule represents a flexible and feasible practice schedule. In a supervised practice setting, however, both physicians in training and instructors have to coordinate their schedules to be able to attend the practice sessions at the same time. The coordination of two or even more individuals’ practice schedules may be challenged by the demanding and busy clinical life at most hospitals. As the results of our study challenge the superiority of a weekly distributed practice schedule (at least for bronchoscopy skills acquisition), a less distributed practice schedule like the one-day distributed practice schedule may be favoured in a practice setting that includes feedback from an instructor, and a weekly distributed practice schedule may be favoured in an unsupervised practice setting.

Our study has several limitations. In this study, bronchoscopy skills were assessed in an experimental setting. Although it has been shown that bronchoscopy skills acquired through simulation training transfer readily to the operating room (17), investigators are yet to show how specific practice interventions transfer to the operating room. In addition, we only assessed performance with simulator-measured metrics. The objective, structured assessments (OSATS) developed for bronchoscopy simulation were deselected due to the lack of opportunity for blinding of the assessors (18) and due to lack of feasibility (19). Another potential limitation is generalisation of results from this study. As the effects of various distributed practice schedules are likely to be task dependent and dependent on the complexity of the task at hand, the results of our study may not be readily generalised to the practice of medical procedural skills that are less complex than bronchoscopy skills learning. Finally, the number of participants was small, although similar in size to previous distributed practice studies, and this may also affect generalisability.

## Conclusions

We found no difference in effectiveness of basic bronchoscopy skills acquisition between one-day distributed practice and weekly distributed practice. As opposed to previous studies, we found no additional effect of distributing the practice beyond a single day. This discrepancy may be explained by the differences in task type and task complexity between bronchoscopy skills learning and learning to suture and learning basic hand–eye coordination tasks associated with laparoscopy. Our findings suggest that the choice of bronchoscopy training practice may be guided by what best suits the clinical practice.
